# Markedly Delayed Presentation of a Psychotic Disorder 10 Years After the First Onset of Symptoms

**DOI:** 10.7759/cureus.57191

**Published:** 2024-03-29

**Authors:** Aoife B O'Reardon, Melissa N Litenski, Melissa Hernandez, Yakov Niyazov, Jadiyer Acosta, Jose Cruz

**Affiliations:** 1 Department of Psychiatry and Behavioral Health, Herbert Wertheim College of Medicine, Miami, USA; 2 Psychiatry, Mount Sinai Medical Center, Miami Beach, USA

**Keywords:** schizo-obsessional component, community awareness, treatment resistance, long duration of psychosis, schizophrenia

## Abstract

Schizophrenia affects 1% of the population, causing chronic debilitating symptoms with largely unknown causes. Structural brain changes and neurochemical alterations are believed to contribute to its etiology. Delayed treatment initiation is a major concern. This case involves a male patient with a decade-long history of psychosis, experiencing isolation, agoraphobia, and paranoid delusions. His situation deteriorated to the point where he lived in a self-imposed physically constraining environment for a year, leading to muscle atrophy and deteriorating health. Delayed help-seeking was driven by insurance concerns, despite prior academic success.

Following extensive evaluation, he received the diagnosis of schizophrenia (first episode, severe), requiring multidisciplinary treatment, including medication adjustments and therapy. This case serves as a poignant illustration of a missed opportunity for early intervention, with treatment initiated only after symptoms became severe. Research indicates that early intervention in schizophrenia is crucial, typically leading to improved outcomes, emphasizing its critical importance.

## Introduction

Schizophrenia is a severe mental disorder that affects approximately 1% of the general population. The natural history of schizophrenia is often chronic and debilitating. Schizophrenia has been associated with a higher unemployment rate (approximately 90% for working-age patients with schizophrenia) and a high prevalence of schizophrenia in homeless people [1,2]. Despite that, schizophrenia remains difficult to treat and its causes remain largely unknown [3]. Research has demonstrated that schizophrenia is a complex mental illness, involving not only neurochemical alterations but also structural changes in the brain [4,5]. These structural changes often become evident on imaging studies over time and may encompass findings such as cortical atrophy and lateral ventricle widening. Howes et al., for instance, demonstrated that these structural changes tend to be more pronounced in patients with a longer duration of untreated psychosis (DUP) compared to those who are diagnosed and treated earlier [5]. Additionally, apart from the structural alterations, there are notable neurochemical changes observed in terms of significant variations in neurotransmitter levels [4].

In the past decade, much interest has concentrated on patients during their first episode of psychosis because of its seemingly pivotal position in determining the later course of illness. DUP is defined as the time from manifestation of the first psychotic symptom to initiation of adequate antipsychotic drug treatment. It has been postulated that untreated psychosis has a toxic effect through unknown neurological and psychological mechanisms and that DUP predicts worse symptoms, both negative and positive symptoms, and poorer social functioning and quality of life [6]. In addition, continued psychosis could lead to chronic profound harm. In a study by Drake et al., DUP emerged as the single strongest predictor of symptom severity after 6-12 weeks of treatment [7]. This study supports the hypothesis that harm incurred by treatment delay is greatest in the early weeks of psychosis, arguing that early intervention in those who have psychotic symptoms is important to prevent treatment delays from limiting recovery across whole populations [7]. Despite strong evidence, there are extreme gaps in the diagnostic measures for schizophrenia, and delay in treatment is still a common concern.

This idea that a longer DUP leads to poorer outcomes has contributed to extensive changes in mental health services worldwide and has attracted considerable research interest in the past years. Therefore, discussing real-life examples that illustrate these effects goes a long way to exemplify patterns that are present but also highlight the detriment of untreated schizophrenia on a person’s life.

## Case presentation

The patient was a 35-year-old male who was brought to the emergency room by law enforcement due to bizarre behavior, including making statements that the family home needed “an exorcism” and expressing nihilistic ideas as to whether he was alive or not.

On admission, he was in a psychotic state characterized by auditory hallucinations, preoccupations of a religious nature, and bizarre delusions. The auditory hallucinations were prominently of a singular male voice demeaning him as well as concerns of turning into the antichrist. His behavior was disorganized, and he was internally preoccupied. In his appearance, he was disheveled, unkempt, malodorous, with skin ecchymoses, and with poor nutritional status. He also had evidence of significant muscle atrophy, and, on examination, had consequent difficulty ambulating.

A collateral history from his parents revealed an illness consistent with psychosis extending over a decade. They reported that the patient had exhibited marked social isolation and agoraphobia and had frequently voiced paranoid delusions of being poisoned.

More expansive social history revealed that for one year he had lived in a bathtub in his parents’ home. However, no effort had been made to obtain psychiatric help for him during that 10-year period of illness. The only reason offered by the family for the delay in seeking treatment was an unfounded concern about insurance coverage for psychiatric treatment. Notably, before the onset of his illness, he had functioned at a high level with a graduate degree in the sciences. With the onset of his illness, he was unable to work and was never employed in his chosen career. Further, he was single and had no known friendships or romantic relationships. Prior history was negative for substance abuse and labs on admission revealed a blood alcohol content of 0% and a negative drug toxicology screen. The family history was negative for psychiatric disorders.

Differential diagnosis

The differential diagnoses in this case included bipolar I disorder, schizoaffective disorder, schizophrenia, obsessive-compulsive disorder (OCD), substance-induced psychosis, and psychosis due to a general medical condition. However, concerning the latter, lab testing, head CT scan, and urine drug screen were all negative.

Diagnosis

In the unit, the patient consistently reported paranoid delusions, auditory hallucinations, and disorganized speech and exhibited negative symptoms, including flat affect, apathy, and alogia. No evidence of elevated mood, mood lability, hyperactivity, grandiosity, increased rate of speech, or flight of ideas was observed that would support a diagnosis of bipolar disorder. A prior history of OCD was reported by his mother, and while he did not manifest compulsions, he did demonstrate intrusive thoughts. These thoughts were of an obsessive nature such as a fear of engaging in conversations with others due to concerns about unintentionally saying something negative or inappropriate. Additionally, he manifested severe anxiety associated with his delusions. Overall, the patient’s clinical presentation was characterized by prominent auditory hallucinations and paranoid delusions. Considering this patient’s presentation in the unit, and his history of chronic psychotic symptoms, along with his profound functional decline from his premorbid high level of functioning, it strongly indicates that the primary diagnosis was schizophrenia.

Treatment

Care for the patient required a multidisciplinary treatment approach, encompassing medical management, physical therapy, occupational therapy, and psychiatric care. Caloric intake was supplemented with a daily nutritional supplement. Concurrently, daily physical therapy was initiated due to the patient’s pronounced lower extremity pain, muscular atrophy and weakness, and functional impairments attributed to his prolonged constricted posture.

Initially, the patient’s management was treatment with risperidone 1 mg BID. Additionally, for breakthrough agitation, haloperidol was administered at 5 mg IM BID on a PRN basis. After 10 days of treatment, his psychosis remained unchanged. He exhibited poor hygiene habits, including reluctance to bathe or interact with peers. Further, his food and water intake were sparse in the context of his response to an auditory hallucination of a male voice directing him that “if you eat or drink, you will become the antichrist.” In an effort to address his continued symptoms, risperidone was titrated to 3 mg BID and perphenazine was added at 4 mg BID and was titrated up to 16 mg BID. Perphenazine was added as a typical neuroleptic to try and improve response.

Ultimately, risperidone was not tolerated in the higher dose due to postural hypotension and was discontinued. Olanzapine was commenced in the third week of his hospital stay and the dose was titrated to effect. Perphenazine was continued as before. During his stay, the olanzapine was eventually titrated to a dose of 30 mg nightly combined with perphenazine 16 mg BID. Lorazepam at 0.5 mg TID was added for anxiety. To address comorbid obsessive ruminative symptoms, sertraline was started at 50 mg QAM with titration to 200 mg daily during his hospital stay. In terms of treatment, medications in this case were tailored and chosen due to the patient’s unique presentation with changes made later in his hospital course when his earlier medication regime proved ineffective despite incomplete optimization. This regime produced distinct improvement over the next two weeks with much improved self-care and engagement with other patients in the unit. He developed insight stating that his voices were figments of his imagination and could be safely disregarded. His paranoia also lessened to some degree.

Follow-up post-discharge

In the initial post-discharge planning, consideration was given to enrolling the patient in a Partial Hospitalization Program. However, it was felt that the patient still lacked sufficient insight to derive significant benefit from this program at that time. Consequently, an alternative plan was formulated, which entailed the implementation of a five-day-a-week social rehabilitation program combined with weekly physical therapy sessions. Additionally, the patient was scheduled for regular follow-up appointments at the outpatient psychiatric center affiliated with the hospital on a biweekly basis.

Regarding his living situation post-discharge, the patient was planning to return home. However, in preparation, the parents received intensive psychoeducation. More specifically, they were counseled about his medication regime, potential side effects, and the importance of life-long aftercare and follow-up. Additionally, the parents were also strongly advised to engage with a lawyer regarding aftercare for the patient should they become deceased.

In terms of current status, the patient continues to attend his outpatient aftercare programs as well as psychiatry appointments. His most recent psychiatric progress note was favorable, and he continues to make stepwise progress.

## Discussion

There were multiple striking features seen in this patient presentation including a prominent schizo-obsessional component and a 10-year interval between the first onset of psychosis and first treatment. The patient experienced intrusive thoughts of an obsessive nature. These thoughts included a fear of engaging in conversations with others due to concerns about unintentionally saying something negative or inappropriate. He also had religious fixations including a fear to eat or drink food because he would go to hell or become the antichrist. This particular feature was very pervasive and the patient had to be prompted to eat and drink water regularly.

Obsessive-compulsive (OC) symptoms within the context of schizophrenia have been documented in various manifestations as an integral component of the schizophrenic experience for more than a century. According to Cavaco et al., having OC symptoms or OCD alongside schizophrenia can contribute to heightened psychopathological severity and a less favorable prognosis [8]. Enhanced diagnostic precision enables tailored interventions through optimized psychotherapeutic and psychopharmacological strategies. However, identifying this concurrent symptomology can be difficult because there can be significant overlap between intrusive OCD thoughts and psychosis-related delusions. Unfortunately, up until now, there has been minimal advancement in comprehending the clinical and neurobiological importance of the OC phenomenon in schizophrenia.

Regarding the extended duration of untreated psychoses, multiple meta-analyses have indicated an association with a range of adverse outcomes at six months. These include outcomes related to total symptoms, overall functioning, positive symptoms, and quality of life. Additionally, it was found that patients with a longer DUP were significantly less likely to achieve remission. It is now thought that shortening the DUP may be a potentially modifiable prognostic factor in schizophrenia [6,9]. What can be done to prevent such delayed presentation of psychosis in the future as happened in this case?

A study by Melle et al. examined the impact of a major public informational campaign in the community on early detection of psychosis and subsequent outcomes [10]. The impact of a public information campaign aimed at the public and schools used a historical control design method to measure the effect with and without the public awareness campaign by looking at the DUP and outcomes in two patient cohorts, historically. The first psychosis cohort (n = 108) was diagnosed with the public awareness campaign in place and the second (n = 75) after the campaign was suspended [10]. Without a public awareness campaign, patients had a longer DUP, more severe symptoms, and worse functioning. It was concluded that without a public awareness campaign, there was a reduction in help-seeking behavior and an increase in the DUP. It is possible, though uncertain, that a greater public awareness of psychosis in our area might have led to earlier help-seeking behavior by the family and earlier psychiatric intervention in this case.

There are two novel aspects to this patient’s medication treatment worthy of note. The dose of olanzapine was titrated to 30 mg daily, beyond the FDA-approved drug range in a relatively short period of treatment. The rationale here was the protracted duration and severity of his psychotic illness in the context of the absence of any improvement with risperidone. Utilizing olanzapine instead of risperidone may also have been more advantageous in this case because of its differing receptor binding profile. In addition, olanzapine was combined with the typical neuroleptic perphenazine in a combination-type treatment approach. The combination of a typical antipsychotic of medium potency, perphenazine, with the atypical agent olanzapine may have provided additional efficacy in this case to attain symptom remission and clinical improvement of treatment-resistant psychosis. The empirical evidence in support of such approaches is limited, though in the case of higher olanzapine dosing, a post hoc analysis did find a dose-response relationship with better responses at higher doses (up to 40 mg daily) for patients with higher symptom severity at baseline [11]. The addition of sertraline was helpful in reducing his obsessional symptoms. Therefore, in unusual clinical circumstances such as these, a creative approach may be in the best interests of the patient.

## Conclusions

This case report highlights the connection between a long DUP and subsequent severity of illness at clinical presentation. In this case, psychotic symptoms were present for a full 10 years before our patient finally presented to the emergency room for evaluation and treatment, at which point he was in a very deteriorated state and no longer able to care for himself. Unfortunately, it appears that a lack of insight, both in the patient and family as a whole, seems to have prevented any distinct efforts to seek help across 10 years of his severe mental illness.

This case also illustrates the role of a multidisciplinary approach in severe illness, with this case needing combined nursing, psychiatric, occupational and physical therapy, social work, and careful discharge planning for treatment to be effective. Pharmacotherapy also had to be both systematic and intensive, ultimately needing a combination of both a typical and atypical neuroleptic with the dose of the latter (olanzapine) escalated above the usual therapeutic range for treatment to be effective in this case.
